# An Attention-Controlled Hand Exoskeleton for the Rehabilitation of Finger Extension and Flexion Using a Rigid-Soft Combined Mechanism

**DOI:** 10.3389/fnbot.2019.00034

**Published:** 2019-05-29

**Authors:** Min Li, Bo He, Ziting Liang, Chen-Guang Zhao, Jiazhou Chen, Yueyan Zhuo, Guanghua Xu, Jun Xie, Kaspar Althoefer

**Affiliations:** ^1^School of Mechanical Engineering, Xi’an Jiaotong University, Xi’an, China; ^2^State Key Laboratory for Manufacturing Systems Engineering, Xi’an Jiaotong University, Xi’an, China; ^3^Department of Rehabilitation, Xijing Hospital, Fourth Military Medical University, Xi’an, China; ^4^Faculty of Science & Engineering, Queen Mary University of London, London, United Kingdom

**Keywords:** hand exoskeleton, hand rehabilitation, brain-controlled rehabilitation, rigid-soft combined robot, EEG

## Abstract

Hand rehabilitation exoskeletons are in need of improving key features such as simplicity, compactness, bi-directional actuation, low cost, portability, safe human-robotic interaction, and intuitive control. This article presents a brain-controlled hand exoskeleton based on a multi-segment mechanism driven by a steel spring. Active rehabilitation training is realized using a threshold of the attention value measured by an electroencephalography (EEG) sensor as a brain-controlled switch for the hand exoskeleton. We present a prototype implementation of this rigid-soft combined multi-segment mechanism with active training and provide a preliminary evaluation. The experimental results showed that the proposed mechanism could generate enough range of motion with a single input by distributing an actuated linear motion into the rotational motions of finger joints during finger flexion/extension. The average attention value in the experiment of concentration with visual guidance was significantly higher than that in the experiment without visual guidance. The feasibility of the attention-based control with visual guidance was proven with an overall exoskeleton actuation success rate of 95.54% (14 human subjects). In the exoskeleton actuation experiment using the general threshold, it performed just as good as using the customized thresholds; therefore, a general threshold of the attention value can be set for a certain group of users in hand exoskeleton activation.

## Introduction

Hand function is essential for our daily life (Heo et al., 2012). In fact, only partial loss of the ability to move our fingers can inhibit activities of daily living (ADL), and even reduce our quality of life (Takahashi et al., 2008). Research on robotic training of the wrist and hand has shown that improvements in finger or wrist level function can be generalized across the arm (Lambercy et al., 2011). Finger muscle weakness is believed to be the main cause of loss of hand function after strokes, especially for finger extension (Cruz et al., 2005; Kamper et al., 2006). Hand rehabilitation requires repetitive task exercises, where a task is divided into several movements and patients are asked to practice those movements to improve their hand strength, range of motion, and motion accuracy (Takahashi et al., 2008; Ueki et al., 2012). High costs of traditional treatments often prevent patients from spending enough time on the necessary rehabilitation (Maciejasz et al., 2014). In recent years, robotic technologies have been applied in motion rehabilitation to provide training assistance and quantitative assessments of recovery. Studies show that intense repetitive movements with robotic assistance can significantly improve the hand motor functions of patients (Takahashi et al., 2008; Ueki et al., 2008; Kutner et al., 2010; Carmeli et al., 2011; Wolf et al., 2006).

Patients should be actively involved in training to achieve better rehabilitation results (Teo and Chew, 2014; Li et al., 2018). Motor rehabilitation has implemented Brain Computer Interface (BCI) methods as one of the means to detect human movement intent and get patients to be actively involved in the motor training process (Teo and Chew, 2014; Li et al., 2018). Motor imagery-based BCIs (Jiang et al., 2015; Pichiorri et al., 2015; Kraus et al., 2016; Vourvopoulos and Bermúdez I Badia, 2016), movement-related cortical potentials-based BCIs (Xu et al., 2014; Bhagat et al., 2016), and steady-state motion visual evoked potential-based BCIs (Zhang et al., 2015) have been used to control rehabilitation robots. However, the high cost and complexity of the preparation in utilizing these methods mean that most current BCI devices are more suitable for research purposes than clinical practices. This is attributable to the fact that the ease of use and device cost are two main factors to consider during the selection of human movement intent detection based on BCIs for practical use (van Dokkum et al., 2015; Li et al., 2018). Therefore, non-invasive, easy-to-install BCIs that are convenient to use with acceptable accuracy should be introduced to hand rehabilitation robot control.

Owing to the versatility and complexity of human hands, developing hand exoskeleton robots for rehabilitation assistance in hand movements is challenging (Heo et al., 2012; Arata et al., 2013). In recent years, hand exoskeleton devices have drawn much research attention, and the results of current research look promising (Heo et al., 2012). Hand exoskeleton devices mainly use linkage, wire, or hydraulically/pneumatically driven mechanisms (Polygerinos et al., 2015a). The rigid mechanical design of linkage-based mechanisms provides robustness and reliability of power transmission, and has been widely applied in hand exoskeletons (Tong et al., 2010; Ito et al., 2011; Arata et al., 2013; Cui et al., 2015; Polygerinos et al., 2015a). However, the safety problem of misalignment between the human finger joints and the exoskeleton joints may occur during rehabilitation movements (Heo et al., 2012; Cui et al., 2015). Compensation approaches used in current studies make the mechanism more complicated (Nakagawara et al., 2005; Fang et al., 2009; Ho et al., 2011). Pneumatic and hydraulic soft hand exoskeletons, which are made of flexible materials, are proposed to assist hand opening or closing (Ang and Yeow, 2017; Polygerinos et al., 2015a; Yap et al., 2015b). However, despite bi-directional assistance—namely finger flexion and extension—being essential for hand rehabilitation (Iqbal et al., 2014), a large group of current soft hand exoskeleton devices only provide finger flexion assistance (Connelly et al., 2010; Polygerinos et al., 2013, 2015a; Yap et al., 2015a, b). Wire-driven mechanisms can also be complex to transmit bi-directional movements since wires can only transmit forces along one direction (In et al., 2015; Borboni et al., 2016). In order to transmit bi-directional movements, a tendon-driven hand exoskeleton was proposed, where the tendon works as a tendon during the extension movement and as compressed flexible beam constrained into rectilinear slides mounted on the distal sections of the glove during flexion (Borboni et al., 2016). Arata et al. (2013) attempted to avoid wire extension and other associated issues by proposing a hand exoskeleton with a three-layered sliding spring mechanism. Hand rehabilitation exoskeleton devices are still seeking to achieve key features such as low complexity, compactness, bi-directional actuation, low cost, portability, safe human-robotic interaction, and intuitive control.

In this article, we describe the design and characterization of a novel multi-segment mechanism driven by one layer of a steel spring that can assist both extension and flexion of the finger. Thanks to the inherent features of this multi-segment mechanism, joint misalignment between the device and the human finger is no longer a problem, enhancing the simplicity and flexibility of the device. Moreover, its compliance makes the hand exoskeleton safe for human-robotic interaction. This mechanism can generate enough range of motion with a single input by distributing an actuated linear motion to the rotational motions of finger joints. Active rehabilitation training is realized by using a threshold of the attention value measured by a commercialized electroencephalography (EEG) sensor as a brain-controlled switch for the hand exoskeleton. Features of this hand exoskeleton include active involvement of patients, low complexity, compactness, bi-directional actuation, low cost, portability, and safe human-robotic interaction. The main contributions of this article include: (1) prototyping and evaluation of a hand exoskeleton with a rigid-soft combined multi-segment mechanism driven by one layer of a steel spring with a sufficient output force capacity; (2) using attention-based BCI control to increase patients’ participation in exoskeleton-assisted hand rehabilitation; and (3) determining the threshold of attention value for our attention-based hand rehabilitation robot control.

## Exoskeleton Design

### Design Requirements

The target users are stroke survivors during flaccid paralysis period who need continuous passive motion training of their hands. They should also be able to focus their attention on motion rehabilitation training for at least a short period of time. For the purpose of hand rehabilitation, an exoskeleton should have minimal ADL interference and have the ability to generate adequate forces to perform hand flexion and extension with a range of motion that is similar or slightly lower than the motion range of a natural finger.

To achieve minimal ADL interference, the device is to be confined to the back of the finger and the width of the device should not exceed the finger width. Here, the width and height constraints of the exoskeleton on the back of the finger are both 20 mm. Low weight of the rehabilitation systems is a key requirement to allow practical use by a wide stroke population (Nycz et al., 2016). Therefore, the target weight of the exoskeleton should be as light as possible to make the patient feel more comfortable to wear it. The typical weight of other hand exoskeletons is in the range of 0.7 kg–5 kg (CyberGlove Systems Inc., 2016; Delph et al., 2013; Polygerinos et al., 2015a; Rehab-Robotics Company Ltd., 2019). In this article, the target weight of the exoskeleton is less than 0.5 kg.

There are 15 joints in the human hand. The thumb joint consists of an interphalangeal joint (IPJ), a metacarpophalangeal joint (MPJ), and a carpometacarpal joint (CMJ). Each of the other four fingers has three joints including a metacarpophalangeal joint (MCPJ), a proximal interphalangeal joint (PIPJ), and a distal interphalangeal joint (DIPJ). The hand exoskeleton must have three bending degrees of freedom (DOF) to exercise the three joints of the finger. For some rehabilitation applications, it is unnecessary for each of the MCPJ, PIPJ, and DIPJ of the human finger to have independent motion as long as the whole range of motion of the finger is covered. Tripod grasping requires the MPJ and IPJ of the thumb to bend around 51° and 27°; MCPJ, PIPJ, and DIPJ of the index finger to bend around 46°, 48°, and 12°; and for the middle finger to bend around 46°, 54°, and 12° (In et al., 2015). For the execution speed of rehabilitation exercises, physiotherapists suggest a lower speed than 20 s for a flexion/extension cycle of a finger joint (Borboni et al., 2016). It has to be stressed that hyperextension of all these joints should always be carefully avoided.

The exerted force to the finger should be able to enable continuous passive motion training. In addition, the output force should help the patient to generate grasping forces required to manipulate objects in ADL. Pinch forces required to complete functional tasks are typically below 20 N (Smaby et al., 2004). Polygerinos et al. (2015b) estimated each robot finger should exert a distal tip force of about 7.3 N to achieve a palmar grasp—namely four fingers against the palm of the hand—to pick up objects less than 1.5 kg. Existing devices can provide a maximum transmission output force between 7 N and 35 N (Kokubun et al., 2013; In et al., 2015; Polygerinos et al., 2015b; Borboni et al., 2016; Nycz et al., 2016).

The design should allow some customization to hand size and adaptability to different patient statuses and different stages of rehabilitation.

### Rigid-Soft Combined Mechanism

**Figure 1:**
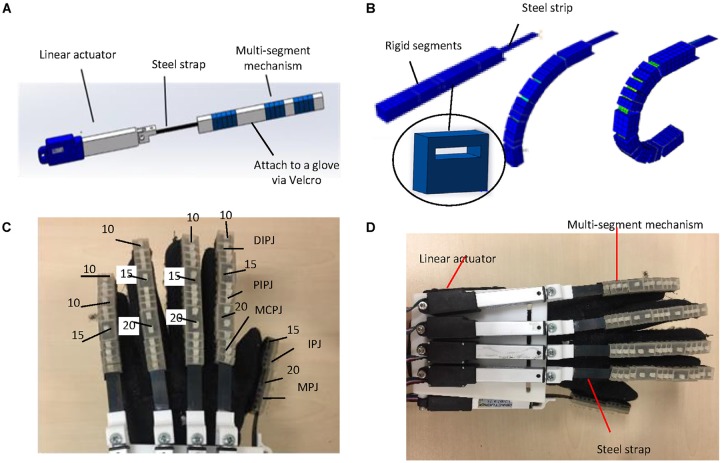
Design of the hand exoskeleton: **(A)** CAD drawing of the index finger acuator; **(B)** bending motion generated by the proposed mutli-segment mechanism with a spring layer; **(C)** segment thicknesses (unit: mm); and **(D)** overview of the hand exoskeleton prototype.

Based on our established design requirements, a hand exoskeleton was designed and constructed (see Figure 1). In our design, each finger was driven by one actuator for finger extension and flexion, resulting in a highly compact device. A multi-segment mechanism with a spring layer was proposed. It has respectable adaptability, thus avoiding joint misalignment problems. A three-dimensional model of a single finger actuator is shown in Figure 1A. This finger actuator contained a linear motor, a steel strap, and a multi-segment mechanism. As shown in Figure 1B, the spring layer bended and slid because of the linear motion input provided by the linear actuator. The structure then became like a circular sector. When the structure was attached to a finger, it supported the finger flexion/extension motion. Five finger actuators were attached to a fabric glove *via* Velcro straps and five linear motors were attached to a rigid part which was fixed to the forearm by a Velcro strap. Each steel strap was attached to a motor by a small rigid 3D-printed part. It should be noted that the current structure is not applicable to thumb adduction/abduction.

Based on the mechanism design, we developed a prototype hand exoskeleton for left hand. The finger exoskeleton actuators can be easily replaced since they are attached to a glove with Velcro straps. The segments of the multi-segment mechanism were made of VisiJet Crystal material using a rapid prototyping machine (3D Systems MJP3600). To attach the linear actuator, the rigid part was made of PLA using a rapid prototyping machine (D3020, Shenzhen Sundystar technology co. Ltd, China). The weight of the prototype is 401 g, including the glove, multi-segment mechanism, and motors, which is much lower than the target weight (0.5 kg). The characteristics of the linear motors are listed in Table 1. The thickness and length of the steel strip are 0.3 mm and 80 mm, respectively. Since the actuator bends more at the joints than at the finger segments, the segments of the multi-segment mechanism above the joints are designed to be thinner than the segments above the distal phalangeal, proximal phalangeal, and metacarpal phalangeal. The segments of the multi-segment mechanism above the joints are 12 mm × 10 mm × 5 mm. Thumb IPJ and MPJ have three and four segments, respectively. There are 3, 3, and 4 segments for DIPJ, PIPJ, and MCPJ of the index, middle, and ring fingers. The numbers are 2, 2, and 4 for DIPJ, PIPJ, and MCPJ for the pinky finger. The thicknesses of other segments (unit: mm) are shown in Figure 1C. Figure 1D shows the overview of the hand exoskeleton prototype.

**Table 1:** Characteristics of the linear motor.

Manufacturer	Firgelli Technologies. Ca
Model	L12-50-210-12-I
Weight	40 g
Stroke	50 mm
Repeated position accuracy	0.2 mm
Max. speed	5 mm/s
Max. side force	30 N

## Brain-Controlled Switch for the Hand Exoskeleton

**Figure 2:**
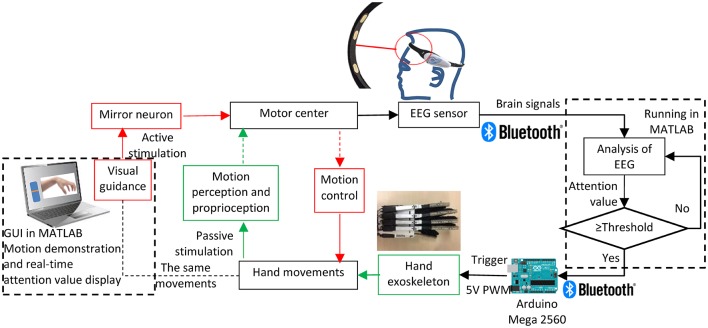
Brain-controlled switch for the hand exoskeleton.

Figure 2 displays a schematic diagram of the brain-controlled switch for the hand exoskeleton. Here, a commercialized Brainlink Lite device (Macrotellect Ltd., Shenzhen, China) with EEG sensors (NeuroSky, Inc., San Jose, CA, USA) was used in the brain-controlled switch for the hand exoskeleton. This device is easy to wear and does not need to apply the conductive gel. It contains a specially designed electronic circuit perceives the brain signals, filters out the noise and the muscle movement, and converts to digital power. EEG sensors are integrated into a headband. There are three dry electrodes in this device including an EEG signal channel, a REF (reference point), and a GND (Ground Point). EEG band power values for delta, theta, alpha, beta, and gamma were recorded with a sampling rate of 512 Hz in the frequency range between 3 Hz and 100 Hz. This device monitors the contact between the electrodes and the skin. If this device fails to collect EEG or receives poor signals, it will issue a warning to notify the user to adjust the sensor.

Mirror neurons, which link vision and motion, can be activated either when an individual acts, mentally practices an action, or observes the same action performed by another human, robotic actions, or virtual characters (Oztop et al., 2013). Moreover, motor imagery can be enhanced based on visual guidance, thus promoting motor recovery (Li et al., 2015; Pichiorri et al., 2015; Liang et al., 2016). Therefore, a motion demonstration is shown on the screen providing visual stimulation to mirror neurons. A graphical user interface (GUI) in MATLAB was designed to display the visual guidance. The user should look at the demonstration on the screen and imagine the movement. The intensity of mental “focus” or “attention” of the user was used as the brain-controlled switch for the hand exoskeleton. The attention value was acquired using the built-in patented Attention Meter algorithm (NeuroSky, 2018) in a ThinkGear AM (TGAM) module (NeuroSky, Inc., San Jose, CA, USA) in this Brainlink Lite device. In the last few years, there were some reports about using the TGAM module and the Attention Meter algorithm in BCI studies (Iordache, 2017; Cui et al., 2018). Unfortunately, the specific attention value calculation function was not possible to find out as it is regarded as a trade secret. But we could guess that the attention value should be calculated from the recorded EEG band power values. The BrainLink device transmitted data to a MATLAB program in a computer *via* a Bluetooth connection. A threshold of attention value was defined to turn on the hand exoskeleton. Once the user reaches the threshold of the attention value, the hand exoskeleton will be activated to conduct the same motion as shown on the screen providing stimulation to the motion perception and proprioception. The real-time attention value is also shown on the screen using a bar. An Arduino MEGA 2560 (Arduino, Ivrea, Italy) was used as the controller of the hand exoskeleton. Bluetooth connection was also used between the Arduino and the computer. The Arduino MEGA 2560 controlled the linear motors using the 0–5V interface mode of the linear motors. The 0–5V input voltage to the motor had a linear relationship to its 50 mm stroke.

## Experimental Setup and Protocols

Experiments were conducted to evaluate the proposed rigid-soft combined hand exoskeleton and the attention value-based switch for the hand exoskeleton. Human subjects were involved in these experiments. The experiments were undertaken in accordance with the recommendations of the Declaration of Helsinki. The study was approved by the Institutional Review Board of Xi’an Jiaotong University. All subjects provided signed informed written consent before the start of the experiments.

### Hand Exoskeleton Characterization

For preliminary evaluations of the developed prototype, we characterized the finger motion output and force output.

#### Motion Control Test

This section describes the evaluation experiment of the range of motion under the actuation of the hand exoskeleton. Two sessions of hand movements were conducted including an active hand motion session (driven by the subject’s hand muscles) and a passive hand motion session (driven by the exoskeleton). The range of motion of the finger joints in these two sessions was compared. A healthy human subject (male, 24 years old, right handed) worn the hand exoskeleton and participated in this experiment. Twenty-one retroreflective hemispheric markers, each 9 mm in diameter, were attached to the finger joints of a healthy subject. An active hand closing (from the open position where finger and palm were straightened to the flexed position) and opening session (from the flexed position to the open position) was conducted in order to obtain the maximum joint angle that the subject can normally actively perform. A passive session was then performed, where actions were performed with the assist of actuators. In this passive session, the subject was instructed to relax the finger, and the exoskeleton performed a hand closing and opening motion. During the process, the finger movement was recorded using a VICON motion capturing system, a 3D motion capture system with 10 T40 MX cameras that can recode at a frequency of 500 FPS (OML Co., UK). The joint angle values during the motion were calculated from the captured position data of the markers.

#### Output Force Measurement

In order to characterize the force output of the hand exoskeleton prototype, two types of output force measurement experiments were conducted. We measured the amount of force received by the fingertips during the exoskeleton actuation, and the maximum pulling force when grabbing an object.

**Figure 3:**
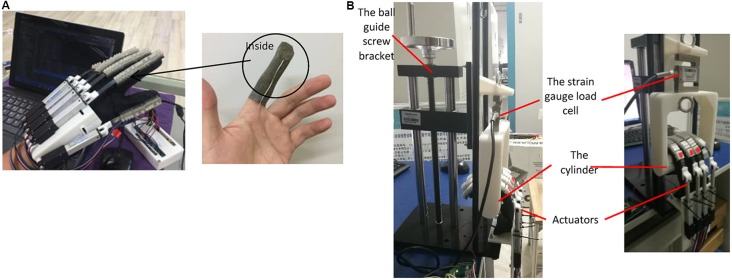
Output force measurement experimental set-up: **(A)** the interactive forces between the exoskeleton and the human fingertips during the exoskeleton actuation; and **(B)** tension provided by the finger exoskeleton in a grasping motion.

For the first type, the interactive forces between the exoskeleton and the human fingertips were recorded during a hand closing and opening process. A Finger TPS System (Pressure Profile Systems, Los Angeles, CA, USA) was used to measure the output force of the finger exoskeleton at the fingertip. The technical characteristics of the fingertip force sensor are listed in Table 2. The experimental set-up is shown in Figure 3A. The same human subject as in the motion control test worn a finger TPS sensor for each finger inside the glove of the hand exoskeleton. The force sensing element was placed between the fingertip and the exoskeleton. The subject’s hand was passively driven by the exoskeleton to conduct a hand closing and opening motion. The TPS System recorded the fingertip forces throughout the process.

**Table 2:** Characteristics of the fingertip force sensor.

Sensor thickness	2–3 mm
Measuring range	22.73 kg
Resolution	0.045 kg
Sampling frequency	40 Hz

For the second type, the tension provided by the finger exoskeleton in a grasping motion was measured with a strain gauge load cell (Model THL-1, Bengbu, China). This sensor has a range of 500 N (sensor error: 0.02% of full scale). The experimental platform is shown in Figure 3B. A cylinder made by 3D printing was connected to a ball guide screw bracket by the strain gauge load cell. The ball guide screw bracket ensured that the cylinder moved up and down smoothly and accurately. Four actuators were fixed at the bottom of the ball guide screw. During the test, the actuators were bent to the maximum extent and the postures were maintained. Simultaneously, the cylinder was slowly pulled by the ball guide screw and moved up. Force data were started collecting when the finger came into contact with the cylinder; the output of the sensor was then recorded every 3 mm cylinder displacement. The experiment was repeated three times.

### Performance of the Brain-Controlled Switch for the Hand Exoskeleton

Experiments were conducted to evaluate the performance of the attention value-based switch for the hand exoskeleton involving human subjects. Fourteen participants, including 10 males and four females, with an average age of 23.8 (SD = 0.89) were involved in the experiment. All the experiments were conducted in a quiet room. Participants were asked to sit in front of a laptop and wear the Brainlink device.

#### The Threshold of Attention Value

The purpose of this part was to determine the threshold of attention value. The experiment measured the attention values of the subject during resting state, during concentration practice with visual guidance, without visual guidance, and with distractions. During experiment of the resting state, the participants were idle and not imagining any movement. This process lasted for 50 s. In the experiment of concentration practice with visual guidance, participants were asked to watch a video clip of a hand grasping motion and focus on the action for at least 50 s. The grasping video played over and over again for the entire 50 s duration. During the distraction experiment, they were asked to listen to the sound of the simulating collision or the noise of the factory while watching three video clips. At the same time, they were asked to answer random questions. During the concentration practice without visual guidance, subjects were asked to look at the blank screen and imagine the action of grasping. No video guidance was provided at this time. At the beginning of each trial, the start of the trial was cued to the participant by a voice prompt. This experiment was repeated three times for each participant, and the attention values were recorded. Before the experiment, the process was explained to each participant and they had one trial to get familiar with the experiment process without recording their data. Between each trial, there was a break lasting at least 1 min.

#### Attention-Based Control of the Hand Exoskeleton

The purpose of this part was to evaluate the proposed attention-based exoskeleton control method by measuring the response time and success rate of the rehabilitation exoskeleton activation based on the threshold measured in the previous section. Since the attention value threshold measured when participants were focusing on the video was higher than the one without visual guidance, visual guidance was applied here. Participants were asked to look at the motion demonstration of hand grasping on the screen and to imagine the same motion. If the threshold was reached, the hand exoskeleton would be activated to conduct the same motion. Each participant repeated this process twenty times, respectively. The response time for each test was recorded. Some subjects need a short period of time to clear their thoughts and focus on the hand motion. On the other hand, it is difficult for the human subjects to maintain a high level of attention value for a long time. Therefore, we set a 30 s time limit for each trial. When the time exceeded 30 s, the test was marked as a failure.

## Experimental Results

### Hand Exoskeleton Characterization

#### Motion Control Test

**Figure 4:**
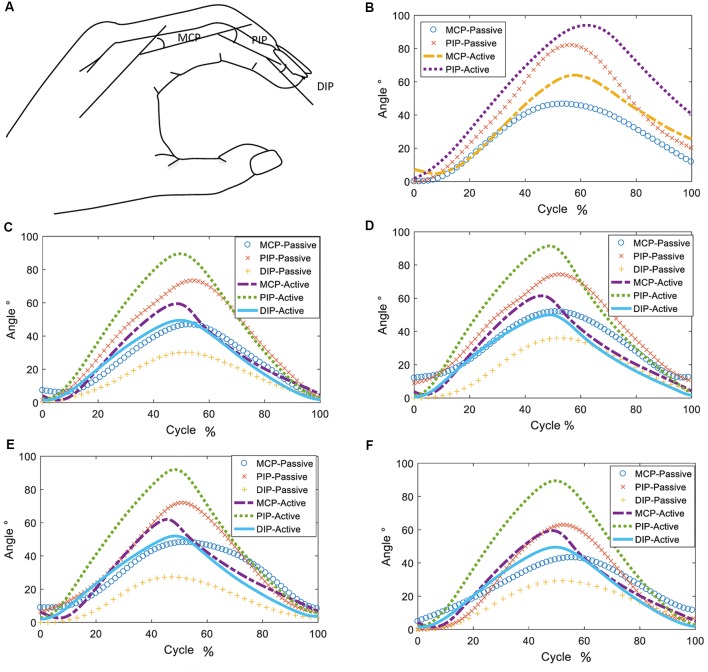
Definitions of joint angles, shown in **(A)** and changes of joint angles during passive and active hand opening and closing: **(B)** thumb, **(C)** index finger, **(D)** middle finger, **(E)** ring finger, and **(F)** pinky finger.

**Figure 5:**
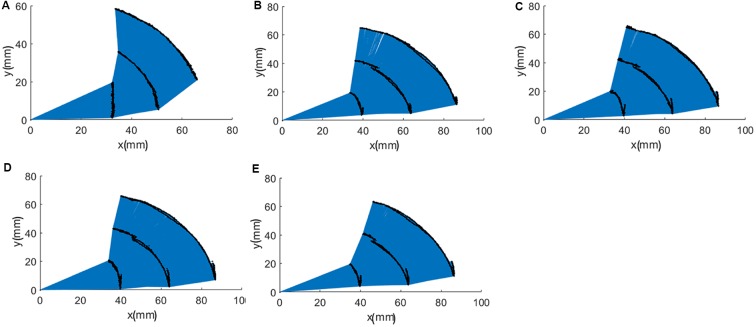
Trajectories of fingertips, distal interphalangeal joint (DIPJ), and proximal interphalangeal joint (PIPJ): **(A)** thumb, **(B)** index finger, **(C)** middle finger, **(D)** ring finger, and **(E)** pinky finger.

Figure 4A shows the definition of the joint angles. The range of motion of a single joint was compared to the maximum joint angle in the active session and the results are shown in Figures 4, 5, as well as in Table 3. Although the proposed finger exoskeleton achieved smaller joint bending angles, the mechanically guided motion of the prototype corresponded well to the active human finger motion. Since the achieved finger joint angles are larger than the finger joint angle requirements of tripod grasping shown in Section II (MPJ and IPJ of thumb bends around 51° and 27°; MCPJ, PIPJ, and DIPJ of the index finger bends around 46°, 48°, and 12°; and the middle finger bends around 46°, 54°, and 12°, respectively), we consider the range of motion of our proposed prototype sufficient for ADL assistance and finger rehabilitation.

**Table 3:** Range of motion.

	Finger	MCP (MP for thumb)	PIP (IP for thumb)	DIP
Active (°)	Thumb	6.72–64.21	1.14–94.17	
	Index	3.10–61.81	2.15–92.35	1.93–50.16
	Middle	2.95–61.86	1.13–91.24	0.68–50.27
	Ring	2.23–60.23	1.47–92.16	1.22–51.58
	Pinky	2.16–62.33	1.82–92.88	1.44–49.31
Passive (°)	Thumb	0.04–46.64	0.02–82.17	
	Index	3.40–47.91	1.55–74.46	1.23–30.13
	Middle	3.01–51.97	3.15–74.35	1.29–36.11
	Ring	2.23–48.27	4.16–72.09	1.22–27.37
	Pinky	3.94–43.31	0.11–62.98	0.05–29.36
Percentage (%)	Thumb	72.64%	87.26%	
	Index	77.39%	80.53%	60.10%
	Middle	83.86%	82.45%	72.06%
	Ring	80.09%	78.28%	53.28%
	Pinky	69.56%	67.73%	59.48%

#### Output Force Measurement

**Figure 6:**
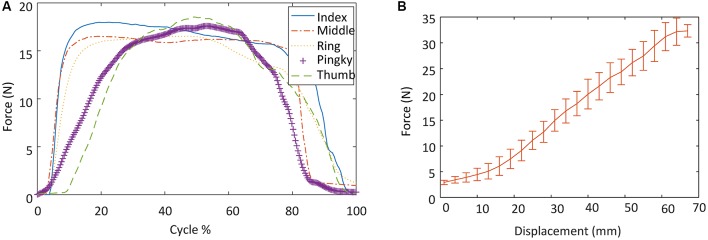
Output force measurement experimental results: **(A)** the interactive forces between the exoskeleton and the human fingertips during the exoskeleton actuation; and **(B)** tension provided by the finger exoskeleton in a grasping motion.

As shown in Figure 6A, during the hand closing and opening process, the maximum interactive force between the exoskeleton and the human fingertips was 17.98 N, 16.52 N, 16.56 N, 17.71 N, and 18.70 N, respectively, for the thumb, index, middle, ring, and pinky finger. The results of the pulling force experiment are shown in Figure 6B. Four fingers can provide up to 30.87 ± 0.97 N of pull force. As described in Section “Exoskeleton Design”, each robot finger should exert a distal tip force of approximately 7.3 N to achieve a palmar grasp, which is four fingers against the palm of the hand, to pick up objects weighing less than 1.5 kg. We consider the output force of the proposed finger exoskeleton acceptable to provide assistance in ADLs.

### Performance of the Brain-Controlled Switch for the Hand Exoskeleton

#### The Threshold of Attention Value

**Figure 7:**
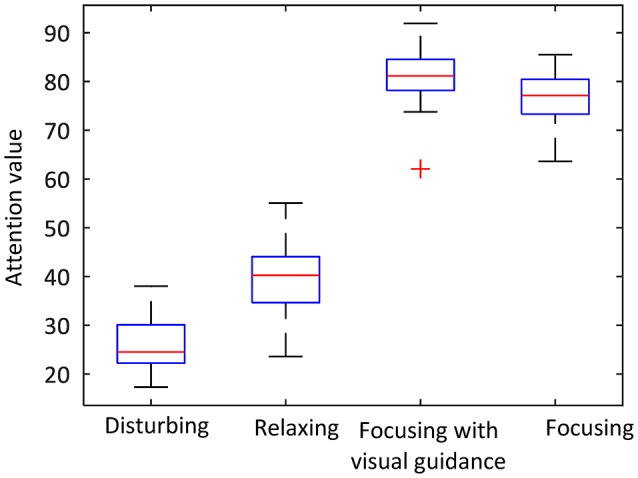
The average attention values in the attention threshold experiment.

An average attention value was calculated for each trial from a 30 s period of data when the attention value reached a stable status. The sample size was 42 (14 subjects × 3 repeats). As shown in Figure 7, the mean attention value of those subjects was 26.3 (SD = 10.7), 40.2 (SD = 10.70), 76.4 (SD = 8.74), and 81.1 (SD = 9.38), respectively, for the experiment of distraction, resting, concentration with no visual guidance, and concentration with visual guidance. A Shapiro-Wilk test was used to check the sample normality. The test results showed that the attention values of those experiment parts all had normal distributions (resting: *W* = 0.988, *p* = 0.925, focusing: *W* = 0.981, *p* = 0.7, disturbing: *W* = 0.951, *p* = 0.067, focusing with visual guidance: *W* = 0.963, *p* = 0.192). A Levene test was used to test the homogeneity of variance. The test results showed that their variances were equal (*p* = 0.734 > 0.05). Therefore, a one-way analysis of variance (ANOVA) test was used to test whether there were significant differences among those groups and among different human subjects. The analysis result showed that there were both significant differences among those groups (*p* = 1.71 × 10^−67^ < 0.05). Then a student *t*-test with Bonferroni correction was applied for pairwise comparisons using R Project for Statistical Computing. The experiment of distraction had significantly less attention value than the other three experiment parts (compared to resting: *p* = 9.31 × 10^−8^, compared to concentration with visual guidance: *p* < 2.2 × 10^−16^, compared to concentration: *p* < 2.2 × 10^−16^). There was also a significant difference between the average attention value of experiment of concentration with visual guidance and that of the experiment of concentration without visual guidance (*p* = 4.96 × 10^−3^). The average attention value of resting had significant less attention value than that of the experiment of concentration with and without visual guidance (*p* < 2.2 × 10^−16^).

#### Attention-Based Control of the Hand Exoskeleton

**Figure 8:**
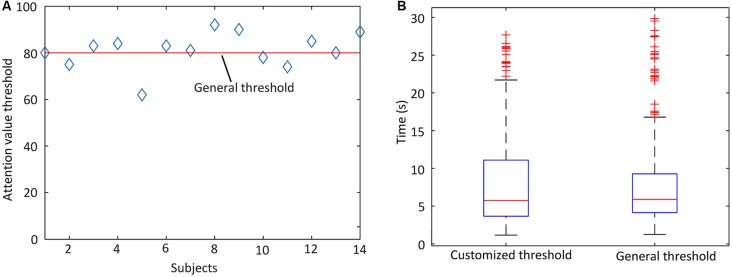
**(A)** Thresholds used in the attention-based hand exoskeleton control experiment and **(B)** the time needed for the participants to reach the attention threshold to activate the hand rehabilitation exoskeleton.

**Figure 9:**
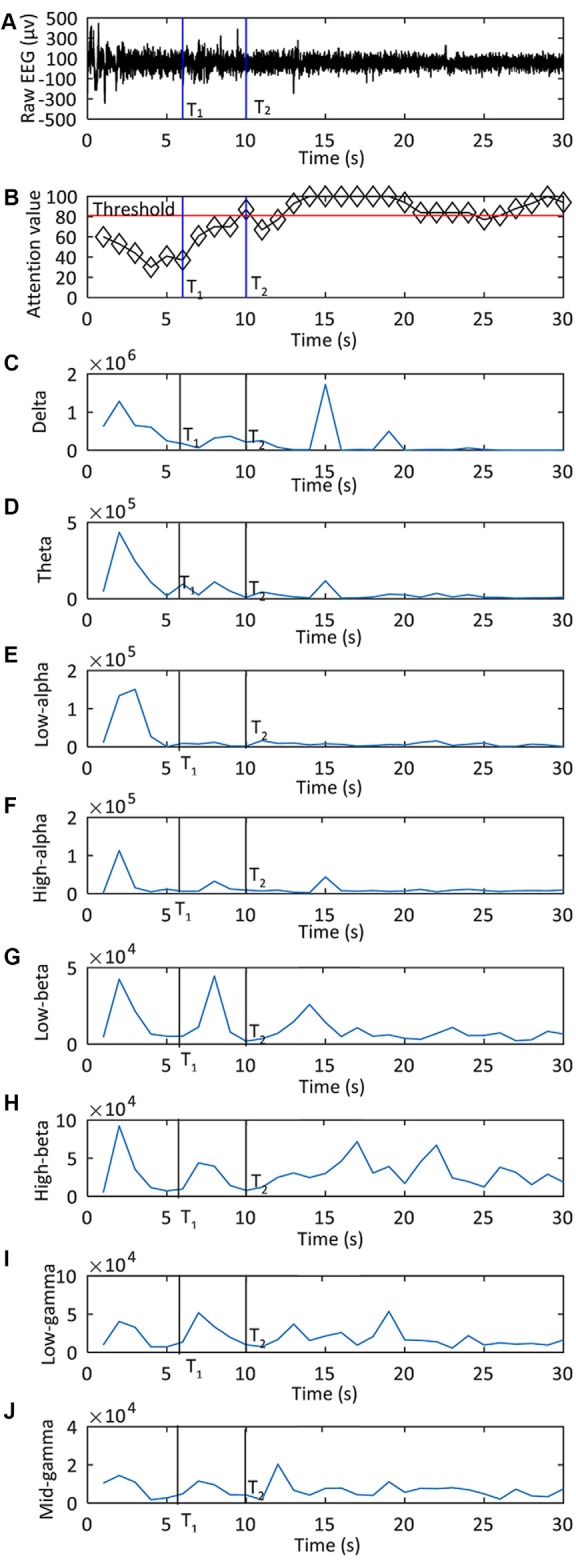
One set of data shown electroencephalography (EEG) signals and attention values extracted from the Brainlink device during the experiment of the attention-based control of the hand exoskeleton: **(A)** the raw EEG signal; **(B)** the attention value used to control the exoskeleton; from **(C–J)** are EEG band power values for delta, theta, low alpha, high alpha, low beta, high beta, low gamma, high gamma, respectively. T_1_ was the moment when the subject heard the voice prompt to focus. T_2_ was the moment when the attention value reached the threshold.

There were 14 subjects and 20 trials for each subject. The attention thresholds used in the experiment are shown in Figure 8A. The hand exoskeleton activation performance of each human subject is shown in Table 4. Figure 9 shows one set of EEG signals and attention values extracted from the Brainlink device. It illustrates the changing process of the attention value from the resting state to the focusing state. T_1_ was the moment when the subject heard the voice prompt to focus. T_2_ was the moment when the attention value reached the threshold. Note that the sampling rate of EEG signals of the ThinkGear AM module in the Brainlink Lite device was 512 Hz (NeuroSky, 2019). However, the attention values were calculated in a rate that could generate one data point per second in the ThinkGear AM module and only one data point every second of the corresponding EEG band power values could be extracted from the software of the Brainlink Lite device. These acquired EEG band power values had no units and were only meaningful compared to each other and to themselves, to consider relative quantity and temporal fluctuations. Error checking was performed against the frame and any frame with errors was discarded. Incidents of data loss over the Bluetooth link were observed in our experiments. During 1 min, about 55–58 data points could be received. Therefore, the data shown in Figure 9 have a time interval of about 1 s between every two data points. Since our threshold-based hand exoskeleton triggering application did not have a high requirement on the accurate time intervals of the attention value data points, data loss of the attention values was ignored for our hand exoskeleton control. The hand exoskeleton had an overall success rate of 94.64% during the experiment with customized thresholds. Using a general threshold, the hand exoskeleton had an overall success rate of 96.43%. In general, the actuation success rate was 95.54%. The ANOVA test result showed that there was no significant difference between the hand exoskeleton activation performance using a generalized threshold and using customized thresholds regarding the actuation success rate (*p* = 0.48 > 0.05). Note that both the actuation success rate using the general threshold and the customized thresholds were not normally distributed (Shapiro-Wilk test: general threshold: *p* < 0.001 < 0.05, customized threshold: *p* = 0.002 < 0.05) and their variances were not equal (Leyene test:* p* = 0.045 < 0.05).

**Table 4:** Hand exoskeleton activation success rates of each human subject (%).

Human subject	Using a general threshold	Using customized thresholds
1	95	100
2	100	100
3	95	95
4	95	95
5	95	95
6	90	100
7	90	90
8	100	100
9	100	100
10	100	100
11	100	70
12	90	85
13	100	100
14	100	95

As shown in Figure 8B, the average time before actuation was 8.06 s (SD = 6.15) and 8.08 s (SD = 6.21) for hand rehabilitation control with a general attention value threshold and with customized attention value thresholds, respectively. A Shapiro-Wilk test was used to test whether the activation time was normally distributed. The test results showed that the time before actuation of each experiment part did not have a normal distribution (hand exoskeleton controlled with a general attention value threshold: *W* = 0.798, *p* < 2.2 × 10^−16^, hand exoskeleton controlled with customized attention value thresholds: *W* = 0.8377, *p* = 5.639 × 10^−16^). Therefore, a Mann-Whitney *U* test was applied to compare the time difference between the two experiment parts. The time difference between the two experiment parts was not significant (*W* = 34734, *p* = 0.5607).

## Discussion

Compared to the hand exoskeleton design in Arata et al. (2013), which has a three-layered sliding spring mechanism, our proposed mechanism has less sliding springs and has a higher output force capacity (30.87 ± 0.97 N pull force for four fingers vs. 3 N per finger). Compared to the hand exoskeleton proposed in Borboni et al. (2016), our proposed design does not have an extra portable trolley carrying an air compressor, pneumatic, or electronic systems. Therefore, the entire device is much smaller.

To optimize the contact force and trajectory, it is necessary to conduct further research on the optimal design of the elements *via* kinematic modeling. Moreover, the actuator should also be adjusted according to different therapy exercises such as strong grasp, hook grasp, clip grasp, and spherical grasp, among others. Since the actuator is attached to a glove with Velcro straps, the actuator can be easily changed.

According to the success rates in the experiment of the attention-based control of the hand exoskeleton, the general threshold performed as good as the customized thresholds. Moreover, there was no significant difference in regards to the time used before the robot actuation in the system evaluation experiment. Therefore, a general threshold of the attention value for a certain group of users can be a good choice in hand exoskeleton activation. In this experiment, only a group of young, healthy people participated. In future studies, a greater number of participants with differing characteristics, such as those who have a medical history involving strokes, should be included.

Linking the intention to execute a movement with sensorimotor feedback has the potential to promote the rehabilitation of stroke patients. In this article, we proposed to use attention value as an input command for the control of the hand exoskeleton. This method is more practical to be clinically used than other complex and expensive BCI methods.

## Conclusion

In this article, we present the design of a brain-controlled hand exoskeleton for the combined assistance and rehabilitation of finger extension and flexion using a multi-segment mechanism. Its compliance makes the hand exoskeleton safe for human-robotic interaction. The device was characterized in terms of its force output and range of motion. The results revealed that the device could achieve hand grasping with acceptable force and motion range. Active rehabilitation training is realized using a threshold of the attention value measured by an EEG sensor as a brain-controlled switch for the hand exoskeleton. In the experiment used to determine the attention value threshold, the average attention value measured when participants focusing on the video motion demonstration was higher than focusing without the visual guidance. An experiment was conducted to evaluate the performance of the attention value-based switch for the hand exoskeleton with visual guidance. The overall activation rate was as high as 95.54%. According to the success rates in the experiment of the attention-based control of the hand exoskeleton, it is evident that the general threshold performed as good as the customized thresholds. Moreover, the time used before the robot actuation in the system evaluation experiment showed there was no significant difference. Therefore, a general threshold of the attention value for a certain group of users can be a suitable choice in hand exoskeleton activation.

For future studies, the multi-segment mechanism will be further optimized to achieve different bending profiles with variable stiffness implemented at different localities. Thus, the device would be highly customizable for different therapy exercises. Moreover, additional experiments with stroke patients are required to further prove the clinical feasibility of the proposed method.

## Author Contributions

ML, JC, and BH: designed the hand exoskeleton. ML and ZL: designed the BCI system. ML, BH, C-GZ, ZL, and YZ: analyzed the data. ML: wrote the article. ML, GX, JX, and KA: contributed materials and analysis tools. KA: language correction.

## Conflict of Interest Statement

The authors declare that the research was conducted in the absence of any commercial or financial relationships that could be construed as a potential conflict of interest.
